# Cancer–malaria: hidden connections

**DOI:** 10.1098/rsob.180127

**Published:** 2018-10-31

**Authors:** Akpéli V. Nordor, Dominique Bellet, Geoffrey H. Siwo

**Affiliations:** 1 Institut Curie, PSL Research University, Département de Recherche Translationnelle, 75005 Paris, France; 2 Institut Curie, PSL Research University, Hôpital René Huguenin, Laboratoire d'Oncobiologie, Pôle Pathologie-Génétique-Immunologie-Hémobiologie, 92210 Saint-Cloud, France; 3 IBM Research Africa, Johannesburg, South Africa; 4 Center for Research Computing, Eck Institute for Global Health, University of Notre Dame, Notre Dame, IN 46656, USA

**Keywords:** cancer, malaria, placenta, genetics, immunology, immunotherapy

## Abstract

Cancer and malaria exemplify two maladies historically assigned to separated research spaces. Cancer, on the one hand, ranks among the top priorities in the research agenda of developed countries. Its rise is mostly explained by the ageing of these populations and linked to environment and lifestyle. Malaria, on the other hand, represents a major health burden for developing countries in the Southern Hemisphere. These two diseases also belong to separate fields of medicine: non-communicable diseases for cancer and communicable diseases for malaria.

Despite the historical divide between cancer and malaria research, evidence accumulated over the past decade points to the need for understanding how the two diseases might influence each other biologically given their evolutionary history and epidemiology. In terms of evolutionary history, exposure of human populations to malaria, especially in Africa, is known to have shaped genetic variation at several loci in the human genome [1]. It is conceivable that if some of the genetic loci under previous or current selection by malaria are also involved in cancer, then this could impact the biology and epidemiology of both diseases. For example, long-term exposure to malaria infection in African populations led to selection of a variant of the Duffy antigen protein associated with reduced susceptibility to malaria [2,3]. While this gene variant evolved to confer protection against malaria, recent evidence suggests that it also influences inflammatory cytokines that are implicated in several cancers [4]. Consequently, this gene variant selected for malaria could also impact cancer outcomes in individuals of African ancestry, including African Americans who are not currently exposed to malaria [4]. Thus, existence of genes that play roles in both diseases could have significance in their biology and epidemiology. With respect to possible epidemiological associations between malaria and cancer, previous studies indicate that the prevalence of malaria is correlated with that of endemic Burkitt lymphoma [5] but negatively correlated with all-cause mortality across multiple cancers [6]. Today, the burden of malaria is decreasing in several countries where the disease has been endemic, while cancer cases are rising in many of those regions, specifically in Sub-Saharan Africa [7]. Here, we give four examples of biological mechanisms with independent studies demonstrating their important roles in both cancer and malaria, and highlight the role of these mechanisms in distinct developmental stages of the malaria parasite. We also discuss how they could impact the clinical management of the two diseases, not only in places where the two diseases co-occur but potentially in all world populations.

The first area where cancer–malaria interactions have been reported is in the human liver. The life cycle of the malaria parasite—*Plasmodium—*involves developmental stages in both the human host and the mosquito vector. In the human host, infection occurs through the bite of female anopheles mosquitoes that inject malaria parasite sporozoites into the human bloodstream. The sporozoites end up in the human liver, where they bind to cell surface proteins of hepatocyte cells, thereby infecting them. In a recent study, *P53*—the most highly mutated gene across several cancers—was shown to play a crucial role in the infection of hepatocytes by malaria parasite sporozoites. Specifically, Kaushansky *et al.* [8] demonstrated that mice expressing increased levels of *p53* had low liver-stage infection by *P. yoelii* while those in which the gene was knocked out experienced a higher parasite burden in the liver. Furthermore, *p53* agonists were more recently shown to eliminate *Plasmodium* liver stage infection in a mouse malaria model [9]. While the role of *p53s* in cancer as an anti-apoptotic protein is widely known, its involvement in malaria highlights the potential for leveraging the results from the vast research on this protein's role in cancer for the discovery of innovative drug targets in malaria. Such research effort would have to start with programmes aiming at deciphering the role of *p53* in human malaria, as initial studies demonstrating its role in the disease have been in rodent malaria [8,9].

The second area where cancer–malaria interactions occur is in the blood stream. Hepatocyte development of malaria parasites takes about 2–10 days in most malaria species, during which infected individuals remain asymptomatic. During this period, sporozoites undergo multiple cell divisions eventually resulting in merozoites, which are released into the bloodstream through the rupture of hepatocytes. Merozoites then infect red blood cells (RBCs) by binding to a number of cell surface proteins and sugars exposed on the outer membrane of these cells. Among these cell surface receptors is the Duffy Antigen Receptor for Chemokines (DARC), which is used by the *P. vivax* malaria species to invade RBCs [2]. The role of this receptor in malaria parasite invasion was first demonstrated by the discovery that many African populations are resistant to infection by this malaria parasite species. It was subsequently discovered that this resistance was due to an inherited polymorphism in the promoter of the *DARC* gene that disrupts its expression specifically in RBCs but leaves its activity intact in other cell types [2]. This explains the low levels of *P. vivax* malaria in Africans when compared with Southeast Asia, where both *P. vivax* and *P. falciparum* infections are common. In addition to its role in malaria, DARC is a decoy chemokine receptor binding both C-C and C-X-C chemokines, but it lacks the capacity to couple to G-proteins, thereby failing to elicit immunological reactions [10]. Consequently, DARC can sequester chemokines in circulation and in the process dampen immunological responses. DARC has been demonstrated to be important in cancer in at least two ways. First, the ability of DARC to sequester chemokines could lower the concentrations of chemokines required for cancer metastasis and tumour neovascularization [11,12]. Indeed, increased expression of DARC in breast cancer cell lines was associated with the inhibition of tumour angiogenesis [11]. Second, DARC interacts with the tumour suppressor protein KAI1 (CD82), leading to the inhibition of proliferation and increased senescence of tumour cells [12]. Recently, the interaction between DARC and KAI1 on macrophages was shown to play a role in the maintenance of dormancy of long-term hematopoietic stem cells, providing an additional mechanism through which DARC may be important in cancer [13]. Therefore, lack of expression of this cell surface receptor on RBCs provides an evolutionary mechanism of protection against malaria infection at the price of having a broader impact on cancers.

Another area where cancer and malaria biology intersect is in relation to immune checkpoint molecules. In cancer, the expression of immune checkpoint molecules presents one of the key mechanisms allowing immune escape underlying the development of tumours. Among these molecules, PD-L1, expressed on the surface of tumours and antigen-presenting cells, interacts with PD-1, expressed on the surface of lymphocyte T cells, and this interaction eventually sends a negative signal to T cells. Over the past few years, public and private research programmes aiming at identifying and developing drugs targeting these immune checkpoint molecules have led to clinical developments and approval of breakthrough anti-cancer immunotherapies. Strikingly, Butler *et al.* [14] recently described PD-1 expression in T cells from children in Mali infected with *P. falciparum*, also suggesting T-cell exhaustion in human malaria. Moreover, they showed that blockade of PD-L1 and LAG-3—another intensively investigated immune checkpoint molecule—restored T-cell function and cleared blood-stage malaria in mice infected with *P. yoelii* [14]. These results thus open an exciting perspective on research on the investigation of anti-cancer immunotherapies in malaria patients, as well as on the exploration of immune checkpoints' gene variants in malaria-endemic regions—where human populations may have been selected for different expression profiles of these genes to resist malaria infection.

The connection between malaria and cancer is also demonstrated in the biology of placental malaria. Malaria in pregnancy is associated with serious complications including low birth weight, stillbirth and spontaneous abortion [15]. While malaria-infected pregnant women may still exhibit the common malaria symptoms, some may be asymptomatic or present with milder symptoms [16]. Malaria parasites sequester in the placenta by using its variant surface antigen (VAR2CSA) to adhere to the placenta [17,18]. VAR2CSA contains six Duffy binding-like domains that bind to chondroitin sulphate A (CSA), a glycosaminoglycan expressed on the placental surface [18]. Interestingly, CSA is also present on the surface of several malignant cells where it is linked to proteoglycans including CD44 and CSPG4 [19,20]. In tumours, CSA enhances aggressiveness and metastatic capacity of malignant cells [19]. The ability of VAR2CSA to specifically bind to the placental form of CSA modification on cancer cells makes it an attractive guide for targeting cancer cells [21]. In line with this, recombinant VAR2CSA (rVAR2CSA) was shown to bind CSA present on the surface of tumours. Subsequently, fusion of diphtheria toxin or conjugation of hemiasterlin derivatives to VAR2CSA inhibits tumour growth and metastasis *in vivo* [21,22]. These results demonstrate how insight gained on a protein that an infectious agent uses to target human cells can be leveraged to target a non-communicable and devastating disease—cancer—and potentially benefit human health.

It is important to note that the unexpected connections between malaria and cancer are not unique to the two diseases but are among those across several diseases at the molecular and epidemiological levels. At the molecular level, it is imperative that as humans have a finite number of genes, many of which are pleiotropic, distinct diseases are bound to interact with the same set of genes. Furthermore, many diseases may perturb similar metabolic and/or immunological processes leading to dependencies between the distinct diseases. At the epidemiological level, disease comorbidities may also arise due to shared environmental risk factors. In this commentary, we focus on malaria and cancer as the two diseases are commonly viewed as fundamentally distinct: whereas malaria is an infectious disease, cancer is non-communicable. It is rare to find collaboration between researchers in the two disease areas or researchers working on the two diseases simultaneously.

The examples provided in this commentary are by no means exhaustive of the connections that may exist between malaria and cancer. However, we have chosen these examples as illustrative for a number of reasons: (i) the connections between cancer and malaria presented here would not have been easily predicted and were discovered serendipitously, (ii) these connections cover distinct developmental stages of the malaria parasite, and (iii) the connections between cancer and malaria considered here show how insights from cancer can be used to find new ways of combatting malaria and vice versa.

The historical divisions between research on cancer and malaria might therefore reflect intellectual constructions rather than biomedical realities. Such artificial silos might negatively impact the way we tackle these two diseases that affect more and more patients worldwide. At the medical level, it might already bias the management of patients affected by malaria and cancer, an increasing problem in developing countries. Hidden links between malaria and cancer also point to potential inefficiencies in the organization of research on diseases. Only a small subset of research projects proactively focus on several types of diseases simultaneously. When it comes to research funding, it is even harder to defend these types of projects in front of funders that generally require that the projects they support only focus on one disease or one disease family. Such organization of care, research and funding might ultimately prevent cross-fertilization in medicine. It makes it even harder for the public to apprehend the high degree of interconnections between human diseases and for researchers to leverage insights across several diseases for the advancement of clinical medicine.

## Data accessibility

This article has no additional data.
